# New Y-SNPs in QM3 indigenous populations of Colombia

**DOI:** 10.1371/journal.pone.0294516

**Published:** 2023-12-06

**Authors:** Marisol Espitia Fajardo, Nelson Rivera Franco, Yamid Braga, Guillermo Barreto

**Affiliations:** 1 Laboratory of Human Molecular Genetics, Biology Department, Universidad del Valle, Cali, Colombia; 2 Research Group in Biology, Languages and History, IMGB, Corpodihva, Mitú, Colombia; Universidad Nacional de la Plata Facultad de Ciencias Naturales y Museo, ARGENTINA

## Abstract

In evolutionary studies of human populations based on the Y chromosome, the majority of Native Americans belong to the QM3 lineage. Therefore, to study the history of groups inhabiting northern South America, it is necessary to have a higher resolution of the tree. The objective of this work was to identify new SNPs of the QM3 lineage that would allow the evaluation of the phylogenetic relationships between Andean and Amazonian populations of Colombia. Sequences previously obtained from two Y chromosomes of Amazonian populations were used, from which 13 potential SNPs were selected and typed in 171 Amazonian samples from the Vaupés region and in 60 samples from the Pasto, Nasa, Embera, Arhuaco and Kogüi ethnic groups of the Andean region. In addition, the main SNPs/markers (L56, L54, M346, M848, Z780, CTS11780) defining autochthonous Q lineages were typed, along with others defined by different SNPs/markers as reported in the literature (CTS11357, SA05, Z19319, Z5915, and Z19384). It was found that all the new SNPs are present in the Amazonian samples and only 2 of them are shared with the Embera, Nasa and Pasto, but none with the Kogüi and Arhuaco from the northern Andes, in the Colombian Caribbean. Combining the 13 variants of the present study with 14 previously reported and using TMRCA, a new QM3 tree proposal is generated. This method makes it possible to increase the number of sublineages of QM3 with a higher resolution and to detect differences between the different populations of Vaupés in the Amazon, as in the case of the Kubeos and Pisamiras, the latter of which is in grave danger of extinction. These new sublineages are useful for microevolutionary studies of the Amerindian populations of South America.

## Introduction

The American continent was one of the last areas to be colonized by humans. Its settlement has been the subject of extensive debate at the level of evolutionary studies for more than 100 years [1–4]. A large number of genetic-population studies have been carried out on Amerindians, revealing some aspects of evolution and the various migrations that have taken place throughout history and prehistory [5–21]. However, questions related to the time of arrival, number of migrations, and migration patterns across the continent continue to be debated in the scientific community. The most widely accepted scenario to date proposes settlement beginning between about 18.000 to 15.000 years ago (kya) [1, 2, 22–25], and entry into the southern cone shortly after [2, 22, 26].

Interest in knowing the ancestry of the American population has led to defining highly polymorphic Y chromosome-specific markers [22, 27–30]. According to Tarazona-Santos et al. [31], the molecular data provided by the Y chromosome are consistent with linguistic and cultural diversity, continental environmental heterogeneity, and paleoecological data in southern Amerindians. Therefore, it has become a very useful tool to study the origin as well as the demographic past of human populations.

The phylogenetic tree of the Y chromosome provides a development to address the issue of population history and movements within geographic territories, especially the colonization of South America, although it is accepted that the ancestors of the Native Americans arrived from Asia, the number of migratory waves, the places of origin and the moment, still represents one of the topics with the greatest debate in evolutionary studies [13, 14, 18–20, 32].

There are two main founding lineages that identify Amerindian populations: Hg C and Hg Q, both of Asian origin [33]. Hg C is almost restricted to North America, while Hg Q, defined by the M242 (C>T) mutation, is found at low frequencies in Europe, East Asia, and the Middle East [34–36], but is present throughout the bi-continental area (South America and North America), with two main Amerindian founding sublineages: Q-M3, identified as indigenous to Native Americans and Siberian Eskimos [35, 37–39], and Q-L54*(xM3) [40]. However, the phylogenetic position of both mutations in the tree of the Y-chromosome haplogroup Q is controversial because the M3 mutation may have originated before the first human settlement in the Americas [37]. Other Q lineages have also been described in the Americas at lower frequencies, such as Q-Z780, which is found throughout the continent but at lower frequencies than Q-M3 [41].

Until a few years ago, most studies for South America only allowed to determine the main haplogroups of the Y chromosome. However, the genomic era and advances in DNA sequencing technologies have made it possible to carry out large-scale analyses, obtaining almost complete sequences of the Y chromosome, which has allowed us to identify some Y SNPs defining Q-M3 sublineages. One of great importance has been described, present at high frequencies and widely distributed throughout the American continent, Q-M848 [41]. Despite the existence of other Y-SNP reports related to new Q-M3 sublineages, their resolution is still weak to explore the history and demography of these groups, mainly due to the low frequency in the number of samples, therefore, it is still premature to make population genetic inferences from these new sublineages [32, 42].

This demonstrates the need to find new SNP-type variants that allow a greater differentiation of haplogroup Q1b1a1a defined by marker M3 or, failing that, haplogroup Q1b1a1a1a1 defined by marker M848. Therefore, the aim of the present study was to provide new clues about the genetic history of the Amerindian populations of Colombia. Here we present a new set of SNPs obtained from two Amerindian Y chromosomes from Amazonia, which allow us to evaluate the phylogenetic relationships between Andean and Amazonian populations from Colombia.

## Materials and methods

### Samples

**Fig 1:**
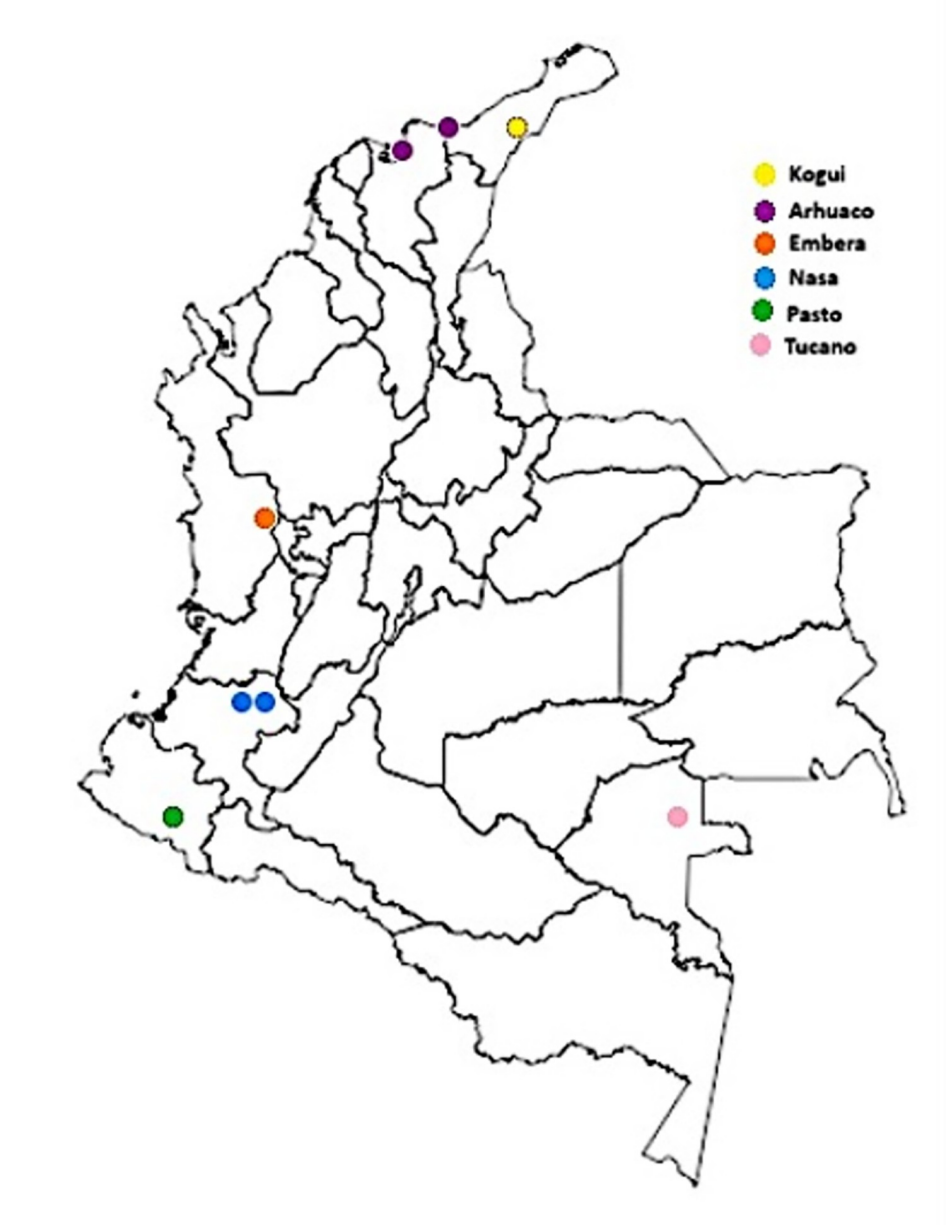
Geographic location of study participants. The colored dots indicate the geographic location of the communities participating in the study.

For this study, a bank of indigenous samples from the Human Molecular Genetics Laboratory of the Universidad del Valle was used, consisting of 207 samples from men belonging to different Amerindian communities in Colombia and assigned to haplogroup Q1b1a1a defined by marker M3. From the southeastern region there were 171 samples from the Tucano-Oriental, Kakua and Hupda communities, and from the Andean region there were 36 samples belonging to the Embera Katio, Nasa (Jambaló, Caldono) and Pasto (Nariño) communities (see Fig 1). All these samples were previously characterized with 17 Y-STRs ((DYS456, DYS389I, DYS390, DYS389II, DYS458, DYS195, DYS385a/b, DYS393, DYS391, DYS439, DYS635, DYS392, GATA-H4, DYS437, DYS438, DYS448) and with 8 Y SNPs (M242, M3, M19, M199, M194, P292 and SA01), which define autochthonous haplogroups of the Y chromosome [19]. In addition, a new sampling was carried out in the Caribbean communities of Colombia, in which 60 samples were collected from unrelated men belonging to the Arhuaco and Kogüi communities (These data are presented in S1 Table). This study was approved by the Human Ethics Committee of the Faculty of Health of the Universidad del Valle, document number 197–010 and 047–021, in accordance with the guidelines of Resolution 8340 of 1993, issued by the Ministry of Health and Social Protection. All samples were obtained with the informed consent of the leaders and each participant. Without exception, sample collection was approved by the Ethics Committee before going out into the field. The Human Diversity Research Line of the Human Molecular Genetics Laboratory of the Universidad del Valle has had ethical approval since before 2007, and this approval is renewed each year according to the current project.

Specifically for this research, the approval of the Human Ethics Committee of the Universidad del Valle was obtained in 2010 (document number 197–010) and is still in force (document number 047–021). No samples or data have ever been collected or used without the prior approval of the Ethics Committee.

The male sample bank, composed of 207 men belonging to different Amerindian communities in Colombia and assigned to haplogroup QM3, was collected during the period 2012–2015. The 60 new samples belonging to men from Colombian Caribbean communities (Arhuaco and Kogüis) were sampled in 2016.

During the period (2012–2019), the data were accessed for research purposes. All sampling was done with the prior informed consent of each study participant and with the approval of the community leaders. This consent was given in writing.

We considered male individuals (our target was the Y chromosome) of legal age, in good health and of sound mind, who voluntarily chose to participate in the study, without consanguinity to the third degree and with at least three generations of Amerindian ancestry. Participants were asked to respond to a brief family history survey about the ethnicity of their parents and grandparents. This was to ensure that individuals were not biologically related.

For participant identification and privacy, each volunteer was assigned a code that identified him or her in all study documents. This code allows for masking of the participant from whom the sample was taken, protection of information, and confidentiality of results.

Personal information (such as name or ID number) that would allow full identification was handled carefully. The researchers have a hard copy and a digital copy of the personal and family information. The hard copy is kept under lock and key at the Human Molecular Genetics Laboratory of the Universidad del Valle. The digital copy was kept in a personal computer used by the researchers for this purpose, with their respective security passwords to prevent access by third parties. The security and responsibility of the data will be the responsibility of the researchers of the project, and for no reason will they be known to third parties, except under legal obligation.

This research does not present any legal risks, since it follows the guidelines of Resolution 8340 of 1993 of the Ministry of Health, which regulates these procedures in the communities. Here we present the survey conducted, the informed consent of each participant, the consent of the leader, and some of the ethical guarantees that were used throughout the research. This will protect the people and the researchers.

### Molecular marker typing

All newly collected samples were typed for eight Y-chromosome SNPs (M242, M3, M19, M194, M199, P292, SA01, and RPS4Y711) by mini-sequencing, using the ABI PRISM® SNaPshot battery. ™ Multiplex System (Applied Biosystems, Foster City, CA; AB); and for 17 Y-STR (DYS456, DYS3891, DYS390, DYS389II, DYS458, DYS19, DYS385, DYS393, DYS391, DYS439, DYS635, DYS392, GATA-H4, DYS437, DYS438 and DYS448), using the Y-Filer kit. The allelic assignment was done with the GeneMapper software to know who belonged to the QM3 haplogroup and how divergent their Y chromosomes were.

### Next Generation Sequencing (NGS) and search for variants of phylogenetic significance (SNP)

In a previous study by Rivera et al. [21], the complete genome of two indigenous samples from the Colombian Amazon was sequenced. From the results of these sequences, only the Y chromosome data were filtered and used to search for new variants of phylogenetic importance. The Variant Annotation and Rsamtools packages of the R software were used to identify the SNPs present in each sequenced sample and not previously reported in public databases such as NCBI (dsSNP) and the 1000 Genomes Project. The variants were manually inspected in the Golden Helix GenomeBrowser [43] program and those variants that were found to be distributed as evenly as possible within the coverage region of the Y chromosome were selected. In addition, only those variants that did not observe repeated regions were considered, indels, or other SNPs close to them, within a 3000 nucleotide field of view, and that were transversions. Thirteen variants were selected (CO15 –CO27).

### Primer design

Allele-specific primers were designed in the WASP web application [44] for each of the 13 preselected SNPs. In addition, primers were designed for the main haplogroups defining autochthonous lineages of Q (L56, L54, M346, M848, Z780, CTS11780) and for some SNPs (CTS11357, SA05, Z19319, Z5915, and Z19384) reported by Jota et al. [45] that were of interest to be classified in the present work according to their origin. Primers were tested in silico using Primer Blast (NCBI). A total of 24 variants were finally typed in the entire Q-M3 indigenous samples included in this study, 13 from the samples sent for sequencing and 11 reported in other studies [45]. See primer sequences in S2 Table.

### SNP genotyping and haplogroup classification

The 24 selected variants were typed by allele-specific PCR in all samples from the communities studied (Tucano, Pasto, Nasa, Embera katio, Kogüi and Arhuaco). In addition to the samples from the Colombian Caribbean (Arhuaco and Kogüi), the 14 SNPs reported by Rivera et al. [21] were typified. The SNPs CO17, CO25, CO26 and Z5915 were typed individually, and the PCR reaction was performed under the following conditions 1X buffer, 4.00mM MgCl2, 0.025mM dNTPs, 0.32μM primers, 0.017U/μL Taq polymerase and 10 ng DNA in a 15μL reaction.

SNPs CO15, CO16 and CO19 were simultaneously amplified in multiplex 001, SNPs CO24, CO27 and CTS11357 in multiplex 002, SNPs CO18 and CO22 in multiplex 003, SNPs CO20 and CO21 in multiplex 004, SNPs CO23 and Z19483 in multiplex 005, SNPs SA05 and Z19319 in multiplex 006, SNPs L54 and M848 in multiplex 007, SNPs M346 and L56 in multiplex 008, and SNPs CTS11780 and Z780 in multiplex 009, using the following amplification conditions 1X buffer, 4.00mM MgCl2, 0.025mM dNTPs, 0.32μM primers, 0.033U/μL Taq polymerase and 10 ng DNA in a 15μL reaction. Thermocycles are shown in S2 Table. The amplified fragments were visualized on polyacrylamide gels and stained with silver nitrate. Haplogroup classification was based on the hierarchical order of the latest Y chromosome phylogeny [46].

### Validation of new Y-SNPs in other Amerindian populations

The new variants reported here were validated in the Amerindian populations of the present study by the absence or presence of the derived allele in each sample. In addition, they were validated in a dataset of modern sequences of the Amerindian Y chromosome belonging to haplogroup Q found in the databases [26, 47], and provaid by [48]. This set consists of 252 males with geographic distribution in Mexico, northwestern Amazon of Colombia, Ecuador, Brazil, Argentina, Bolivia and Peru. In each of these sequences, the position of the variants included in our study was searched to determine if they were present or not. This was done manually using the IGV software [49]. The sequences were in BAM format.

### Genetic structure and population relationships

The AMOVA statistical test was performed with the program Arlequin Ver. 3.5.2.2. [50]. The analysis was performed considering only the own samples typified in the present study. For this, two regions (Amazonian and Andean populations) and three regions (group 1-VpA, group 2-VpB and Andean populations) were considered. VpA and VpB are the two samples whose whole genomes were sequenced. Selected from a network of Y-STR haplotypes from Amazonian populations (belonging to Eastern Tucano language family) whose haplotypes were as distant as possible from each other and located on the distal branches of the network within the expansion nodes, to include as much variation as possible (Fig 3, populations yellow).

To associate the SNPs with the samples and study populations and to observe how they are related, a multiple correspondence analysis (ACM) was performed in the R program version 3.5.3 [51]. The analysis took into account all the SNPs evaluated, and the origin of the samples was used as an additional variable. Due to the large number of Amazonian ethnic groups, they were grouped as group1-VpA and group2-VpB to facilitate the visualization of the results.

In addition, the geographic distribution of the reported subhaplogroups of 483 Amerindian Y chromosomes belonging to the Q-M3 haplogroup was evaluated. Of this subclassification, 231 were performed on their own samples, while the remaining 252 were performed on samples from other studies [26, 47, 48]. (see S1 Table).

### Haplotype network

To observe the behavior of the haplotypes of each of the studied SNPs and to observe their frequency and variation, Y-chromosome haplotype networks were constructed using the Network Ver. 5.0.0.1 software [52]. Network Publisher Ver. 2.1.1.2 (Fluxus-engineering, 2016) was used to output and visualize the networks. The DYS385a and DYS385b loci were not considered due to their duplication and uncertainty in allele assignment. Haplotypic networks between individuals were constructed using the median joining algorithm [53], assigning a weight to each microsatellite as proposed by Muzzio et al. [54].

### Phylogeny and dating

Two Y chromosomes belonging to haplogroup Q-M3 were used for phylogenetic analyses. The new SNPs of this work were included, those reported by Rivera et al. [21] and those described in other studies and present in this investigation. For its construction, the parameters defined by the Y Chromosome Consortium [46] were used, which include the haplogroup hierarchy and nomenclature.

The tree version proposed by [21, 41] was considered as a base, to which the new variants observed in the present study were added. SNPs and subhaplogroups were considered valid if they fulfilled the ISOGG inclusion criteria [46].

To estimate the time to the most recent common ancestor (TMRCA), the calculation of the Rho network statistic was used considering four different mutational rates: 1) EMR (evolutionary mutation rate) considering a mean effective mutation rate of 6.9*10-4/locus/25 years as proposed by [55]; 2) OMRS (observed mutation rate) by [56]; 3) OMRB by [57]; and 4) OMRG by [58]. The ancestral haplotype was inferred using the modal allele of each STR [59], cited by [45]. The above, following the methodology described by Rivera et al. [21]. Considering that dating calculations can vary depending on the amount of information in the sequences, only information from the two complete sequences was used for this estimation. Sequences provided by other authors were only used to validate the variants in this work due to the heterogeneity of the data.

## Results

### Y-STR and Y-SNP in samples from the Colombian Caribbean

Out of a total of 60 samples collected for the Colombian Caribbean, only 24 were classified in QM3. With the addition of these new samples to the existing sample bank at the Human Molecular Genetics Laboratory of the Universidad del Valle, the present study included 231 Amerindian samples.

### SNP genotyping

24 SNPs were typed (13 new and 11 reported in the literature) for the populations studied and, in addition to the samples from the Colombian Caribbean, 14 SNPs reported by Rivera et al. [21], the S4 and S5 Tables describe the position of each of these SNPs. All variants have been named in the document with the prefix "CO", following the order by Rivera et al. [21]. Additional, the S6 and S7 Tables describe the frequency of each of the new Y-SNPs reported here and those typified from the literature.

SNP CO23 and SNP CO20 presented the lowest frequency in the study (2.1%), both are found in the most distal branches of the proposed phylogenetic tree, SNP CO23 was restricted to the Desano and Tukano populations of the linguistic subfamily Tucano-Oriental, while SNP CO20 is exclusively restricted to the Kubeos population. Our language classification is based on [60].

The SNPs CO24 and CO27 presented the highest frequency, they were found in 153 (66.2%) of 231 individuals, they also have a wide distribution, with the exception of the Caribbean populations and the Nasa, they were found in the rest of the populations. SNP CO15, SNP CO19, SNP CO22, SNP CO17 and SNP CO26 have relatively low frequencies and are restricted to the Amazon region, mostly associated with the Tucano and Pisamira populations. On the other hand, SNP CO16, SNP CO18, SNP CO21 and SNP CO25 have higher frequencies, although they are also restricted to Amazonian samples, especially to the Kubeos population, where they have the highest frequency.

The SNPs that define autochthonous haplogroups for Amerindians, Q-L56, Q-M346, Q-L54, Q-M848, were present in all study samples and, as expected, the frequency of SNPs Z780 and CTS11780 was null in the study.

With the exception of the SNPs that define major Amerindian haplogroups (Q-L56, Q-M346, Q-L54, Q-M848), the other variants studied in this research had a null frequency for the Colombian Caribbean samples. Regarding the five variants reported by Jota et al. [42], their frequencies were null for all samples typed in this study.

### Validation of SNPs in other study populations from the literature

From the information reported by Arias et al. [48], data were used from a total of 227 Amerindian samples from the northwestern Amazon belonging to Hg Q-M3, from the work by Pinotti et al. and Mallick et al. [26, 47] used information from 16 and 9 samples, respectively. In both cases, the Q-M3 samples were from different locations in South America. Here, a total of 38 SNPs were validated, including both new ones and those reported by other authors [21, 45], with the aim of analyzing their behavior and distribution in different populations.

Regarding the autochthonous Amerindian haplogroups (Q-L56, Q-M346, Q-L54, Q-M3), as expected, 100% of the analyzed samples, that is 252, belonged to these lineages. The sublinaje Q-M848 showed a high frequency, it was found in 147 individuals (58.3%), the SNPs CO10, CO12 and CO24 showed relatively high frequencies, they were found in 59 individuals (23.5%), they also show a high distribution, they were found in samples from Brazil, Ecuador and Colombia. The SNPs CO03 and CO05 were found in 39 individuals (15.5%), the SNP SA05 in 12 individuals (4.8%) from the Colombian Orinoquía. The SNPs CO01, CO02, CO08, CO13, CO14 and CO24 were also found at lower frequencies. It should be mentioned that several of the sequences did not have data in the position where each SNP was found. When we have the complete sequences of the Y chromosome region, it is very likely that the frequency of reported SNPs will increase. See the details of this validation in the S8 Table.

### Genetic structure and population relationships

Analysis of Molecular Variance (AMOVA) shows significant differences between the study populations; when comparing the Andean populations with the Amazonian ones, it was possible to observe that the highest percentage of variation contributed between regions (53.89%), followed by variation within populations (46.31%), and finally between populations within groups was close to zero. With the exception of the source of variation between populations within groups, in all cases there was statistical significance of each component of variance (Table 1). When the Amazonian samples were considered as 2 groups (Group1-VpA, Group2-VpB) in the analysis and compared with the Andean populations, the percentage of variation between regions decreased (45.94%), the level between populations within groups increased (20.88%) and within populations decreased slightly (33.18%) (Table 2). These percentages of variation are due to the way the populations were grouped.

**Table 1:** Molecular analysis of variance (AMOVA) two groups (Amazonian and Andean region).

Source of variation	D.F.	Sum of squares	Variance components	Percentage of variation
Among regions	1	392.612	3.01475 Va	53.89944*
Among populations within groups	8	11.997	-0.01172 Vb	-0.20959*
Within populations	222	701.959	2.59026 Vc	46.31015*
**Total**	231	1106.568	5.59328	100.00

*

p<0.001. Note: Populations refer to ethnic groups.

**Table 2:** Molecular analysis of variance (AMOVA) three groups (Group 1, Group 2 and Andean populations).

Source of variation	D.F.	Sum of squares	Variance components	Percentage of variation
Among groups	2	392.612	2.59093 Va	45.94*
Among populations within groups	7	208.706	1.17801 Vb	20.88*
Within populations	222	505.250	1.87130 Vc	33.18*
**Total**	231	1106.568	5.64023	100.00

*p<0.001. Note: Populations refer to ethnic groups.

**Fig 2:**
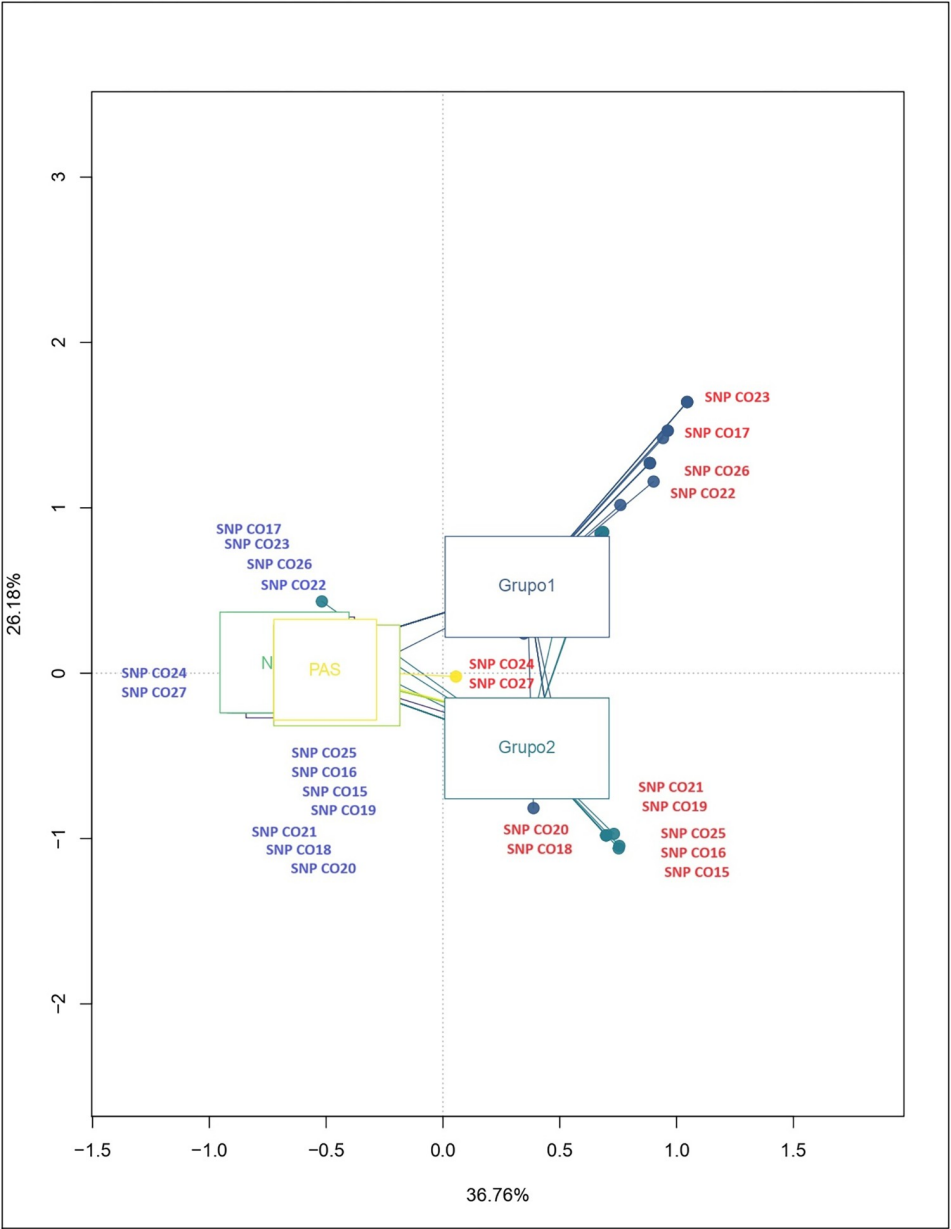
Multiple correspondence analysis by geographic group. The samples are grouped into nodes according to the geographical location to which they belong. Groups 1 and 2 belong to the Amazonian populations and the other group formed belongs to the Andean populations (Pasto, Nasa, Kogüi, Arhuaco and Embera-Katio). Three main groups of SNPs were observed in both the ancestral (blue) and derived (red) states. The SNPs in the derived state were grouped as follows: 1) SNPs CO22, CO26, CO17 and CO23 are closely clustered and related to group 1; 2) SNPs CO15, CO16, CO25, CO19 and CO21 are at the same node and correlated with geographic group 2. SNPs CO24 and CO27 occurred at the same node, in an intermediate position between the two previous groups. In contrast, the SNPs in the ancestral state were related to the Andean groups.

Fig 2 shows the multiple correspondence analysis by SNP, which allowed to explain 62.94% of the total variation, the different SNPs are correlated with their respective geographical locations. It is possible to observe the formation of three main groups. The SNPs CO15, CO16, CO25, CO19, CO21, CO18 and CO20 are related to group 1; the SNPs CO22, CO26, CO17 and CO23 correlated with group 2 and the SNPs CO24 and CO27 occurred in a node in the middle of the two previous groups (group1-group2). Groups 1 and 2 are restricted to samples from the Amazon region, while the other group formed shares some samples from the Andean region (Embera Katio and Nasa Caldono).

The analysis was also carried out in terms of linguistic groupings. However, the separation of the two Amazonian groups was not clearly observed (all samples are from the Tukano family). For this reason, we keep the analysis based on geography.

The classification of the subhaplogroups evaluated in the present work in 483 Amerindian Y chromosomes belonging to the Q-M3 haplogroup is presented in S3 Table.

### Y-STR haplotype network for Q-M3

**Fig 3:**
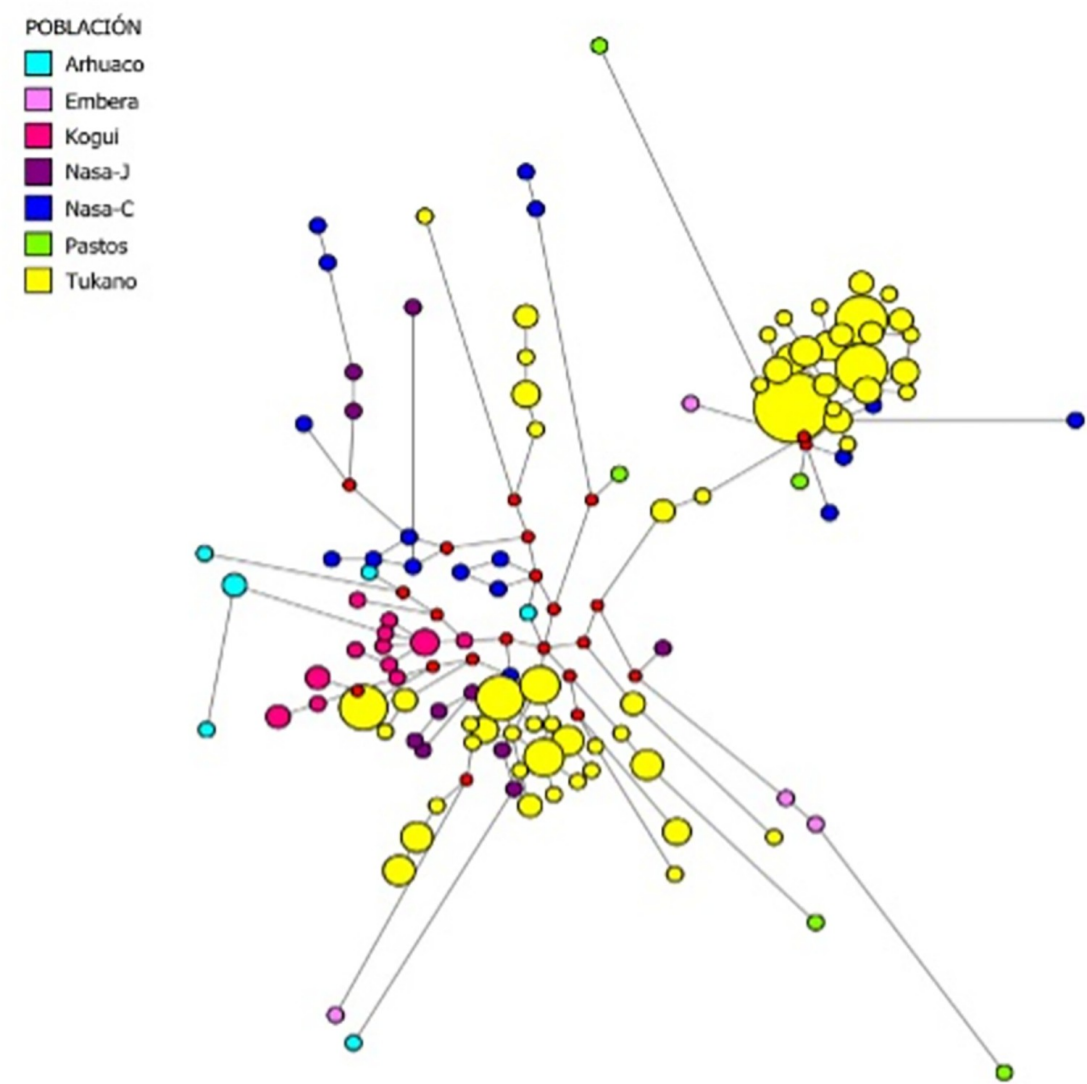
Y-STR haplotype network for Q-M3. The size of the circles indicates the frequency of the haplotype and the length of the lines connecting them are proportional to the mutation steps between the haplotypes. The colors indicate the population of origin.

Reconstruction of the Q-M3 haplotype network (see Fig 3), based on the Y-STRs of the samples typed in the present study, clearly distinguishes two groups of Tucano-Oriental, while the individuals Nasa, Embera, Pasto, Arhuaco and Kogüi are more dispersed in the network. Some Tucano individuals are also dispersed and separated from the expansion nodes formed by the rest of the individuals of the same population group. It is important to emphasize the similarity of some haplotypes of the Andean populations Nasa-C, Embera, Kogüi and Pasto to the haplotype node of the Tucano-Oriental (upper right part of Fig 3), in several cases only one or a few mutational steps away from them.

### Time to the Most Recent Common Ancestor (TMRCA)

Table 3 reports the time to the most recent common ancestor (TMRCA) estimates for each SNP using the Rho statistic. TMRCA estimates for each SNP using the Rho statistic were calculated using two rates of evolution. The EMR estimates gave significantly higher values than those obtained with OMR, with values ranging from 426.38 years for the most recent SNP (SNP CO20) to 14191.43 years for the oldest SNPs (SNP CO24 and SNPCO27). The general pattern of TMRCA with OMR for each SNP was similar to that found with EMR, but its values were between 109.03 and 141.15 years for SNP CO20 and between 3897.80 and 5209.56 years for SNPs CO24 and CO27.

**Table 3:** TMRCA with Rho.

SNP	EMR		OMRS		OMRB		OMRG	
	TMRCA	SD	TMRCA	SD	TMRCA	SD	TMRCA	SD
**CO24, CO27**	14191.43	3639.86	3897.80	933.91	4959.70	1188.34	5209.56	1248.21
**CO15**	7460.88	1993.54	1914.30	511.50	2435.83	650.85	2558.54	683.64
**CO16, CO25**	7168.00	1866.63	1839.15	478.93	2340.21	609.41	2458.10	640.12
**CO19**	7089.31	1798.85	1799.02	454.94	2278.32	578.42	2369.92	615.97
**CO21**	4956.65	1754.20	1271.77	450.09	1618.25	572.71	1699.77	601.56
**CO22**	4840.62	1763.80	1242.00	452.55	1580.37	575.84	1659.98	604.85
**CO18**	4487.92	1719.04	1151.51	441.07	1465.22	561.23	1539.03	589.51
**CO26**	2026.67	1835.22	520.00	470.88	661.67	599.16	695.00	629.35
**CO17**	429.18	319.89	110.12	82.08	140.12	104.44	147.18	109.70
**CO23**	428.02	301.12	109.89	81.78	137.54	102.32	144.56	105.38
**CO20**	426.38	289.32	109.03	81.04	134.35	100.32	141.15	102.32

Note: Time estimates and standard deviation (SD) are in units of years.

### Phylogenetic tree of haplogroup Q-M3 and new subhaplogroups

**Fig 4:**
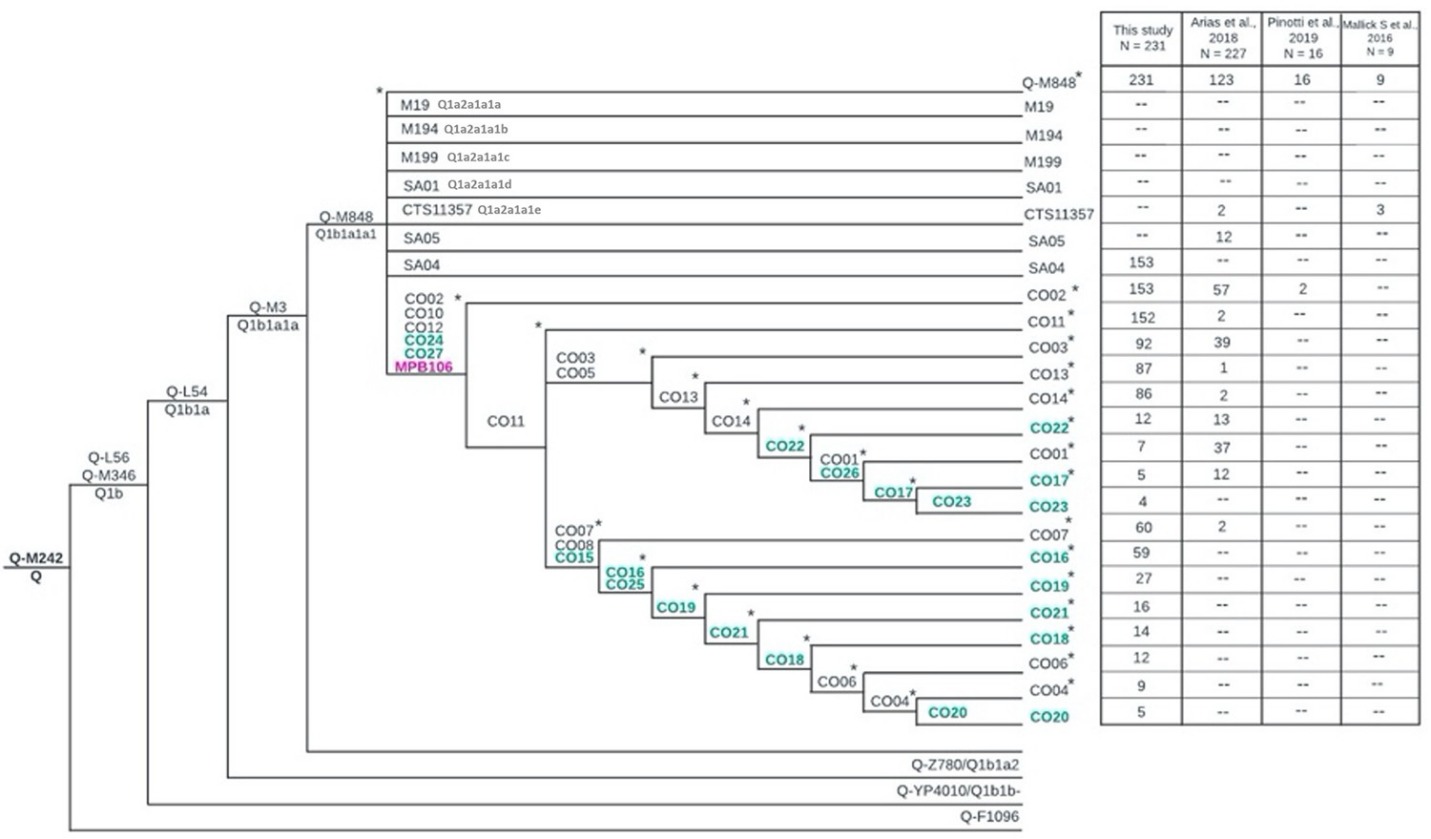
QM3 phylogenetic tree. The new variants described in this work are shown in blue, those of other authors in black [21, 45], and in fuchsia is the variant described by Paz-Sepulveda et al. [2], two of its positions are equivalent to CO10 and CO27.

The results of SNP genotyping allowed the detection of 13 new variants. The phylogenetic tree of Q, including the new variants discovered in the present study and those described by other authors, is shown in Fig 4.

## Discussion

With this study, our laboratory continued the work by Rivera et al. [21] related to the identification of new lineages on the Y chromosome in indigenous populations of Colombia. In this research, the characterization of 13 new Y-SNPs was achieved, which contribute to the knowledge of the genetic diversity of the Y chromosome in Native Americans. In addition, previously reported subhaplogroups with phylogenetic significance within Q were typed, allowing the proposal of a new version of the phylogenetic tree of the Y chromosome that includes highly useful variants for population-level studies. Compared with other studies of new lineages of the Y chromosome, the 13 variants reported here have a high frequency, making them a very useful tool for expanding the phylogenetic repertoire of haplogroup Q-M3, the main lineage among Native Americans [36].

### Differentiation of Amerindian groups in the Amazon region

The newly reported variants show a distribution almost limited to populations from the Amazon. This makes it possible to identify differences between these populations, as in the case of the Kubeos and Pisamiras, the latter in serious danger of disappearing (currently there are only about 20 active speakers and passive speakers are below 60) [61]. In the Amazon region, several indigenous communities coexist with different languages, each with its own characteristics, but where the continuous exchange of practices, knowledge and values persists. This favors the emergence of transethnic identities in which the language can be preserved or modified [62]. The acquisition of these cultural and social practices not only risks the disappearance of some groups, but also produces changes in the patterns of genetic diversity. This is the case of the Pisamira, in addition to the biological loss, an effect of linguistic absorption takes place, by a majority group, which in this case are the Kubeos [61, 62]. Finding their own variants for each ethnic group is very useful for the recovery of their biological identity as a group.

### SA05 sublineage

According to the results by Jota et al. [45] and the origin of their samples, 5 Y-SNPs reported there were typified in the present study; Q-Z5915, which was found in 2 individuals of 272 samples from the Amazon, Q-Z19483 in 60 individuals of 323 samples, most of them Andean from Peru and Bolivia, Q-Z19319 in 21 individuals of 231 Andean samples, Q-CTS11357 in 8 individuals of 272 samples from the western Amazon, and Q-SA05 in 60 individuals of 667 samples from the western Amazon of Bolivia and Peru. When these variants were typed in our study samples, the frequency was zero.

Considering that Q-SA05 appears to have a relatively ancient origin (TMRCA ~10,000 years ago) and that, in addition, previous studies showed a strong association with Amazonian indigenous populations of several linguistic families [45], it was expected to have a wider distribution and to be found in at least a small number of the Amazonian samples in our study. However, this was not the case, and it could be assumed that it is a variant with a restricted geographic distribution, due to genetic drift that is much stronger in Amazonian groups than in Andean ones.

However, the same variant was found in 12 samples belonging to the departments of Meta, Guaviare and Amazonas in Colombia, when it was evaluated in the sequences of samples of indigenous people from the northeastern Amazon in the work by Arias et al. [48]. It could be assumed that there is a gene flow of populations from the western Amazon with a contact route through the Colombian Orinoquía, which would explain the absence of this variant in the Vaupés. Although, it is necessary to increase the number of samples at points far from these regions to determine their behavior and establish what could be happening.

### CO12, CO27 and MPB016 sublineages

The CO12 sublineage was one of the oldest SNP type variants described by Rivera et al. [21] and CO27 one of the oldest new variants of the present research, both from the sequencing of two Y chromosomes belonging to the Eastern Tucanos of the Colombian Amazon. These two sublineages correspond to two of the sites reported by Paz-Sepulveda et al. [2] for the new sublineage MPB016. They are variants of great phylogenetic interest, since they are widely distributed throughout the Amazon region and in some samples from the Andean region typified in this study, a total of 153 samples out of 231. In addition, these variants are also present in the data reported by other authors. In the study by Arias et al. [48] they were found in 57 out of 227 individuals; in the study by Paz Sepulveda et al. [2] and Pinotti et al. [26], they were found in an Ecuadorian of the Cañari ethnic group and in a Brazilian of the Hupda ethnic group. The above is evidence that we are dealing with ancestral variants with a wide distribution in South America, shared by different ethnic groups, which can be very useful for the ancient reconstruction of several of these groups.

### Sublineage Q-CTS11357

This is a relatively old lineage, according to the literature, around 11.3 kya it presented a population focus in Mexico, which eventually spread to the southwestern United States, Central America, reaching Colombia and part of the Brazilian Amazon [2]. In the present study, it had a low frequency, being found in only 5 samples out of a total of 483, belonging to the Achagua-Piapoco ethnic group from Colombia, 2 Nahua-Pima from Mexico and one Karitiana from Brazil. The Achagua-Piapoco belong to the Arawak linguistic family and are mainly found in the department of Meta, in the Colombian Orinoco region. However, their distribution is much wider and Piapoco communities can be found in the departments of Guaviare, Vichada and Vaupés [63].

These populations coexist with communities of different languages, such as the Tucanos, where there is a continuous exchange of practices, values and knowledge. This favors the emergence of transethnic identities in which language can be preserved or modified, in other words, processes of "Arawakization" or "Tucanization" occur [62]. Based on the above, and given its geographic proximity to populations in the northeastern Amazon, we expected this variant to be present in some of the samples from this region. However, its frequency was null, which allows us to consider that perhaps, in addition to language, geography and rivers play a very important role in the expansion, distribution and genetic structure of ethnicities [61, 64].

The Piapoco and Achagua indigenous peoples come from an expansion from the mouth of the Negro River to the Isana and Guainia river basins [63], that is, they present a semi-isolation with the eastern Tucano communities of Vaupés, which could explain the absence of this variant in these populations. However, more samples from both Orinoquía and Vaupés would help to clarify and explain the distribution of this subhaplogroup.

### Variants shared with Andean populations

The 13 new SNPs evaluated show a distribution almost restricted to Amazonian populations, which was expected given the origin of the variants. However, some of them were found in samples from the Andean region (Embera, Nasa-C, Pasto). It is important to note that these Andean populations are linguistically close, belonging to the Chibcha-Paezan branch, while the Tukano are linguistically distant. This result supports the conclusion of the study by Barbieri et al. [19], in which it is mentioned that the genetic structure of South American indigenous populations, if any, is largely disconnected from the linguistic and geographical relationships of the continent. Due to all the social, demographic, environmental, and cultural dynamics that indigenous populations have experienced, it is very difficult to establish a model that can be replicated for the entire continent.

This result could be due to possible gene flow or a common ancestor. With regard to the possibility of gene flow, there could be migration of individuals from the Colombian Amazon to the Andean region. Jota et al. [45], based on Y-STR data, suggest a possible migration route from the Amazon to the Andes for the Q-SA04 lineage, since some of these haplotypes were found on the border between Brazil and Colombia, which could indicate that the Amazonian groups assimilated into the indigenous communities of the central Andes (Quechua and Aymara), as previously suggested by [42], probably during the formation of the Andean states that culminated in the Inca empire. However, according to the results of the Andean and Amazonian Y-STRs evaluated in the work by Rivera et al. [21], a migration from the Amazon to the Andes is unlikely.

If, however, we consider the main entry points to South America to be the Panamanian isthmus and the Darien area in northern Colombia, then it is possible that the populations were split or separated. Some migrated and settled in the Andes, and others migrated toward the Amazon. By then, both populations had some of the older mutations proposed here; this would indicate a common origin of all ethnic groups, with subsequent expansion and linguistic and biological divergence.

Recently, Delgado M et al, [65] conducted a paleogenetic study using mitochondrial genome data from Andean populations of Colombia (Sabana de Bogota), covering individuals from the Late Pleistocene to the early Late Holocene, together with other published data from the region. With the aim of studying the genetic continuity or discontinuity in these populations, the presence of mitochondrial haplogroups A2, B2, C1b, D1 and D4h3a and a reduction in genetic diversity over time were obtained as results. These results show a certain continuity and genetic structure different from those reported in previous works, in which a strong genetic differentiation suggested by mtDNA and a distribution with differences in terms of reported haplogroups are observed.

This could be attributed to demographic processes such as assimilation/mixing between local and extra-regional populations rather than gene flow, and to sex-biased migrations closely linked to changes in social organization [66]. This may be an explanation for the presence of Amazonian variants in Andean samples.

### Amazonian and Caribbean regions not related

The frequencies of the 13 new variants typed in the present study and of the 14 reported by Rivera et al. [21] were zero for the samples belonging to the Caribbean region (Arhuacos-Kogüis). This allows another possible scenario: there was probably a first settlement route along the Pacific coast and an alternative settlement route through the Caribbean island chain, which may have been bidirectional at different times in history [7]. This result supports the hypothesis that there were at least two [8, 67] or three [68] waves of immigration of the first settlers of the Americas. Based on the studies by Keyeux et al. [8] and Keyeux & Usaquen [9], we would speak of two founding populations at the end of the Pleistocene, one of which arrived from Central America through the Isthmus of Panama and colonized the northwestern region of Colombia. The second ancestral population arrived from southern North America through the Antillean island chain and colonized the southeastern region, consisting of the Amazon and Orinoquia regions [69]. However, it is necessary to increase the number of samples in order to draw certain conclusions.

### TMRCA

The TMRCA for each SNP showed differences in the values obtained, due to the mutation rate used. This indicates that the choice of mutation rate is very important, since it depends on the estimation being as close to reality as possible. There are two mutation rates reported as the most used, the genealogical or observed rate (OMR) and the evolutionary mutation rate (EMR), although they have a general pattern, the values estimated with EMR are considerably higher compared to those obtained with OMR.

To study major haplogroups, it is suggested to work with the OMR mutation rate, especially when lineages coalesced to the Neolithic or more recent times [70]. In view of the above, the oldest subhaplogroups reported here have a maximum emergence time of about 5000 years, which coincides with the time of emergence of agriculture in South America. In a study by Goldberg et al. [71], the spatiotemporal patterns of South American populations were analyzed, showing that the population of the continent did not occur in only one expansion, but in two, the first with a period of rapid spread across the continent, followed by a second local expansion in a favorable ecological niche, characterized mainly by a sedentary lifestyle supplemented by hunting with crops and domestication of animals, approximately 5000 years ago, which agrees with the estimated times of the last common ancestor for the populations of this study.

On the other hand, it could be said that the demographic events that were promoted by the diffusion of agriculture could explain the expansion of the other subhaplogroups reported here, whose estimated age ranges between 109 and 2560 years; that is, the most recent SNPs within the phylogeny of haplogroup Q [72].

Our results give us an idea of the relationship between the different sublineages of haplogroup Q, indicating that the oldest SNPs (SNP CO24 and SNPCO27), found in the basal branch of the rest of the lineages, are found in most populations of the study (with the exception of the Nasa-J, Arhuaco, and Kogüi), strengthening the hypothesis of a possible common ancestor in these populations and favoring more than one expansion during the population of the Americas [69]. However, these Y-STR-based date estimates and distance-based statistics should be interpreted with some caution, given that there are multiple mutation rates, and no single one has been defined as the most likely to date. This also changes the interpretations given.

### Y chromosome—linguistic and cultural diversity

The colonization of South America is not clear. According to Tarazona et al. [31], there is a geographic structuring of molecular diversity in these populations, which is related to their history and population structure. Accordingly, there is a biogeographic pattern of differentiation that includes two major dispersal regions, the Andean region (west) and the Amazon region (east). In the Andean region, migration would have been from north to south, with constant genetic flow, large population sizes, and lower levels of genetic structuring, whereas in the Amazon region, dispersal would have followed the coasts and rivers, with populations having less genetic flow, smaller population sizes than the Andean populations, and high levels of population structuring. These authors also noted that the geographic differentiation of genetic variation is also consistent with linguistic and cultural diversity, as well as the environmental heterogeneity of South America and paleoecological data [31].

Although this model holds true for some populations, this pattern cannot be replicated for all South American natives. In subsequent studies, the genetic variation of the Y chromosome lacks a clear structure that can be related to geography and linguistics at the continental level. This means that a correlation between genetic and geographic/cultural/linguistic structure can only be expected under very specific conditions [73].

This may be due to variations in the markers used or to non-homogeneity in the populations sampled. And where it would be key to keep in mind the elements that have a direct effect on the genetic structure of a population, we have cultural, environmental, demographic, etc. factors. For example, sex-biased migration (i.e., with unequal numbers of males and females), which is very common and can be associated with multiple causes ranging from cultural practices to environmental components. This makes it difficult to decipher local demographic histories and thus to replicate models of population structuring [65].

Another factor that needs to be taken into account is the change in post-marital residence patterns, in which we find patrilocality and matrilocality, which have a direct impact on genetic diversity and structure [65–66].

Taking the Colombian indigenous population as an example, not all of them have the same behavior at the structural level, so it is good to emphasize two main groups: Amazonian indigenous communities and Andean indigenous communities. Based on these two divisions, for the Amazonian populations it is expected that the linguistic component will have a greater weight on their population structure and diversity, since in this area marriages are performed between speakers of different indigenous languages (linguistic exogamy). This increases genetic diversity through a particular genetic flow (asymmetric) in which women are the ones who migrate and men remain settled in their territories [14], which in turn directly affects population structuring.

Regarding the Andean populations, they behave completely different from the Amazonian indigenous communities, where the geographical position is very important in structuring the populations. Among their geographical positions are the central, eastern and western mountain ranges, which act as a physical barrier to gene flow in the different populations [74]. It is also important to mention that the Andean populations have a different social organization than those of the Amazon, they do not have linguistic exogamy, which gives greater weight to geographical proximity, speaking in terms of population structuring.

According to the above, there are many factors that must be taken into account when following a model to try to interpret the distribution of genetic variability in the native populations of South America, among them we have the geographical proximity, linguistic, economic, social differences, sexually biased migrations, residence patterns and, in general, everything that encompasses their social dynamics.

### AMOVA

The AMOVA results are different from those commonly found in Native American populations, where most of the variation is found within populations. In the present research, this variation is being accounted for between regions/groups. In order to understand these results, several aspects need to be considered: 1. The Amazonian origin of the samples that were sequenced and from which the new SNPs that were typed in the rest of the populations were selected, 2. The way in which the populations were grouped within the study and 3. There is no homogeneity between the number of samples for each study region, with by far the largest number of samples from the Amazon region.

When comparing the diversity of the Vaupés groups with other Native American groups, it is possible to see that the difference depends a lot on the region. Mesoamerican and Andean groups tend to have moderate to high diversity values in Y chromosome and mitochondrial DNA. While in Amazonian populations whose social structure is based on small family groups, diversity tends to be moderate to low but with greater differentiation between groups [61, 75].

The local social dynamics of these groups have shaped the genetic diversity and structure we see today. For example, exogamy is practiced in this region, which is undoubtedly one of the main differentiating forces between groups, both from a linguistic and Y-chromosomal point of view. They also have a patrilineal social organization, in which married men continue to live in their father’s territory and the identity of the individual is determined by the ethnolinguistic identity of the father [48, 61].

In addition to social organization, wars, forced displacements, the physical landscape, geographic barriers, subsistence strategies, disease, shamanism, and extinction are factors that alter the dynamics of these Amazonian populations and, therefore, their structure.

## Conclusions

The methodology used in the present study provided 13 new SNPs, which facilitated the proposal of a new version of the phylogeny of Q-M3, which provides an informative male ancestry with new knowledge for research on the history of the peopling of South America.

The absence of these variants in the indigenous peoples of the north coast of Colombia allows us to propose the existence of at least two migratory routes to South America. However, the genotyping of a larger number of samples from this region will allow further clarification of this matter.

The Andean samples show a clear structuring of the paternal lineage, but it is not fully explained by the SNPs reported in this study, therefore it is necessary to search for new variants typical of the Andean region that will allow the evaluation of phylogenetic relationships between these groups. To do this, a larger population sample is needed in Colombia and neighboring regions to reveal its true distribution among native South Americans.

## Supporting information

S1 Table Subhaplogroup classification and geographic distribution of samples. (XLSX)

S2 Table Primers for the characterization of the new SNPs by allele-specific PCR. (XLSX)

S3 Table Time and temperature for SSP-PCR genotyping. (XLSX)

S4 Table Posición of new SNPs. (XLSX)

S5 Table Position SNPs others authors. (XLSX)

S6 Table Absolute frequency of the 13 new SNPs evaluated by ethnic group. (XLSX)

S7 Table Absolute frequencies of the 11 SNPs reported by other authors and evaluated by ethnicity. (XLSX)

S8 Table Absolute frequencies of the 23 SNPs evaluated in other ethnic groups by other authors. (XLSX)

S1 Questionnaire Inclusivity in global research. (DOCX)
