# Supplementary material for: New Y-SNPs in QM3 indigenous populations of Colombia
Source: PLoS One. 2023 Dec 6;18(12):e0294516. doi: 10.1371/journal.pone.0294516 (PMC10699635; doi:10.1371/journal.pone.0294516)
Supplement: S1 Questionnaire — (DOCX) [file pone.0294516.s009.docx]

Inclusivity in global research

PLOS’ policy on inclusivity in global research aims to improve transparency in the reporting of research performed outside of researchers’ own country or community and ensures that PLOS publications reporting global research adhere to high standards for research ethics and authorship. Authors of relevant research articles may be asked to complete the questionnaire below, which outlines ethical, cultural, and scientific considerations specific to inclusivity in global research. This questionnaire may be requested when researchers have travelled to a different country to conduct research, if research uses samples collected in another country, research with Indigenous populations or their lands, or if research is on cultural artefacts. Researchers travelling to another country solely to use laboratory equipment will not normally be required to complete the questionnaire. However, the questionnaire can be requested at the journal’s discretion for any submission – if you have been requested to complete this questionnaire by the PLOS journal you submitted to, please do so.

Please complete the questionnaire below and include this as a Supporting Information file with your manuscript. Note that if your paper is accepted for publication, this checklist will be published with your article in the supporting information files. Please ensure that you reference the checklist in the main body of your manuscript. We suggest adding a subsection ‘Inclusivity in global research’ to your Methods section and adding the following sentence: “Additional information regarding the ethical, cultural, and scientific considerations specific to inclusivity in global research is included in the Supporting Information (SX Checklist)”

The questions have been designed to be applicable to a wide range of study types, and there are subsections for both human subjects research and non-human subjects research. If any of the questions are not relevant to your research please mark them as “N/A” as appropriate.

**Ethical considerations, permits and authorship**

*This section is applicable to all research types.*

Provide details as to who granted permissions and/or consent for the study to take place in the Methods section of your manuscript. This should include the names of **all** ethics boards, governmental organizations, community leaders or other bodies that provided approval for the study. If individuals provided approval refer to these people by their role or title but do not list their name(s).

Reported on page number: 6 and 7

If there were any deviations from the study protocol after approval was obtained please provide details of these changes in the Methods section of your manuscript.
Did this study involve local collaborators that are residents of the country where the research was conducted or members of the community studied? If you do not have any authors from said communities, please provide an explanation for this below.

Reported on page number: There were no changes to the protocol after ethical approval.

All samples were taken by Colombian researcher (from Universidad del Calle, Cali, Colombia). All samples were obtained with the informed consent of the leaders and each participant. Without exception, sample collection was approved by the Ethics Committee before going out into the field.

Everyone listed as an author should meet PLOS’ criteria for authorship and all individuals who meet these criteria should be included in the author byline, rather than the acknowledgements. For further information please see the journal’s Authorship Policy.

**Human subjects research (e.g. health research, medical research, cross-cultural psychology)**

Did you obtain written informed consent from a representative of the local community or region before the research took place? How did you establish who speaks for the community? Details of written informed consent obtained from study participants should be reported separately in the Methods section of your manuscript.

Project researchers traveled to the sampling sites to contact indigenous leaders. The objectives, motivations, risks, benefits, scope, and ethical considerations of the project were presented to them. A copy of the project was left with them so that they could meet with their communities and conduct the appropriate consultations. The researchers then contacted the leaders again to obtain their consent. With this consent, we visited each community and talked with the people, always accompanied by the leader, we talked about the project, read the informed consent form and clarified any doubts. Those who volunteered to participate were given the appropriate sample and survey data.

This mechanism was repeated for each of the communities living in the different regions. The information provided to the participant was the information contained in the project, but in a non-technical language so that it could be easily understood. The researchers were responsible for collecting the information and obtaining informed consent, which was done with care and respect for their customs, given the vulnerability of these populations.

How did members of the local community provide input on the aims of the research investigation, its methodology, and its anticipated outcome(s)?

The community members were key to the development of the research, they listened to us and gave us the opportunity to present the project, and in turn most of them decided to participate. Some community members served as guides and translators (in most communities they spoke the native language).

When engaging with the local community, how did you ensure that the informed consent documents and other materials could be understood by local stakeholders?

First, community leaders were contacted and the project was explained to them in non-technical language so they could easily understand it. Then they were responsible for socializing with the community to see how feasible it was for them to participate. Once the communities agreed to the project, we traveled to the sites to conduct the sampling, and if there were any doubts, we resolved them with the help of the leaders who translated the questions and the answers.

Will the findings of the research be made available in an understandable format to stakeholders in the community where the study was conducted (e.g. via a presentation, summary report, copies of publications, etc.)? Please provide details of how this will be achieved.

The return of the results to the communities has already taken place. We personally visited the communities again and shared the results with them through talks and a presentation. A summary of the research results is provided in a document to the leaders of each community.They also shared some of their culture and customs with us. This exchange of knowledge and experience between communities and researchers is very gratifying.

**Non-human subjects research using specimens/ animals collected as part of the study, or those housed in archival collections. Examples include archaeology, paleontology, botany and zoology.**

Did the permission you obtained from a local authority to perform the study include an agreement on access to outputs and benefit sharing? This may include procedures to enable fair distribution of the benefits and resources arising from the research performed. Please include any details of Prior Informed Consent and Benefit Sharing Agreements obtained. These may be required by field-specific regulations, for example the Convention on Biological Diversity (CBD) and the associated Nagoya Protocol.

NA

If the material used in your study was imported, please A) provide the year it was imported and B) indicate whether permits were obtained to import/export the materials used, C) provide details of any permits obtained. If this information is not available, please indicate this.

NA

If you used archival specimens, please state how the material used in your study was acquired by the institute it is held in and provide details of any permits obtained for the original excavations/ sample collection. If this information is not available, please indicate this.

NA

How was the potential cultural significance of the materials collected in your study to local communities considered in your research design? Were Indigenous peoples and/or local researchers and institutions involved with archaeological excavations / collection of specimens? If so, please provide a description of their involvement.

NA

If your manuscript includes photographs of human remains please indicate whether authors obtained permission from descendants or affiliated cultural communities to do so.

NA
